# Imaging features of adrenal ganglioneuroma: a case report

**DOI:** 10.1186/1756-0500-7-791

**Published:** 2014-11-07

**Authors:** Anass Mohammed Majbar, Sanae Elmouhadi, Mouna Elaloui, Mohamed Raiss, Farid Sabbah, Abdelmalek Hrora, Mohamed Ahallat

**Affiliations:** Clinique Chirurgicale C, Ibn Sina Hospital, Rabat, Morocco; Radiologie Centrale, Ibn Sina Hospital, Rabat, Morocco; Clinique Taza, BP1020, Taza, 35000 Morocco

**Keywords:** Ganglioneuroma, Adrenal tumors, Magnetic resonance imaging, Computed tomography, Diagnosis

## Abstract

**Background:**

Adrenal ganglioneuroma is a rare tumor constituting 20–30% of all ganglioneuromas. It is a benign tumor and can present diagnostic problems when confused with other adrenal solid tumors.

**Case presentation:**

We herein report a case of adrenal ganglioneuroma in a 28-year-old Arabic patient and emphasize the diagnostic role of cross-sectional imaging modalities (computed tomography and magnetic resonance imaging).

**Conclusion:**

Imaging of adrenal ganglioneuromas is diagnostically challenging. Differentiation between adrenal ganglioneuroma and other solid adrenal tumors can be difficult. However, some suggestive features on computed tomography and magnetic resonance imaging are helpful in achieving a correct diagnosis.

## Background

Adrenal ganglioneuroma (GN) is an uncommon benign neuroblastic tumor comprising Schwann cells and ganglion cells [1, 2]. It is a rare tumor, constituting 20–30% of all GNs. Differential diagnosis between adrenal GN and other adrenal solid tumors is difficult. We herein report a case of an asymptomatic adrenal GN in a 28-year-old man and emphasize the diagnostic role of cross-sectional imaging modalities.

## Case presentation

**Figure 1:**
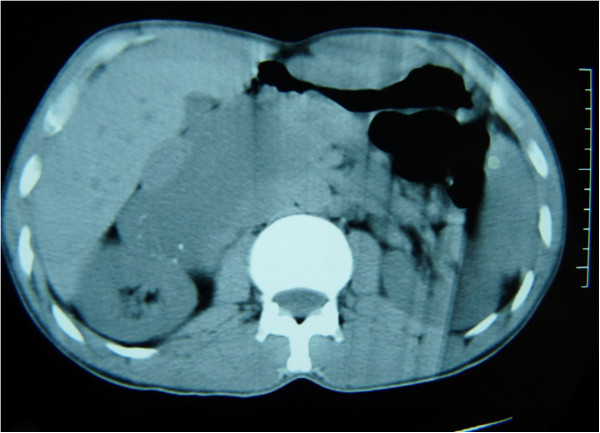
**CT without contrast showing an adrenal tumor with calcifications.**

**Figure 2:**
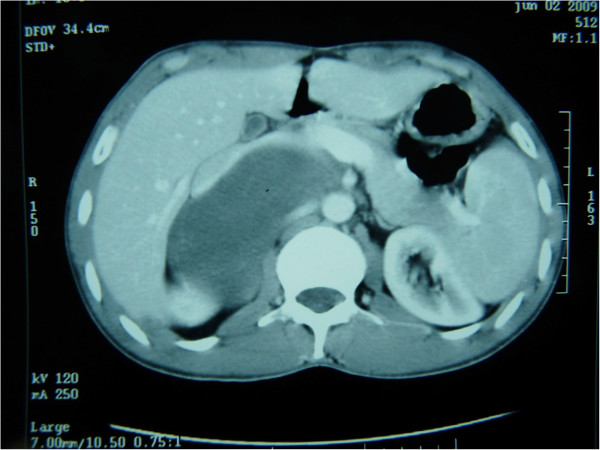
**CT after contrast enhancement.**

**Figure 3:**
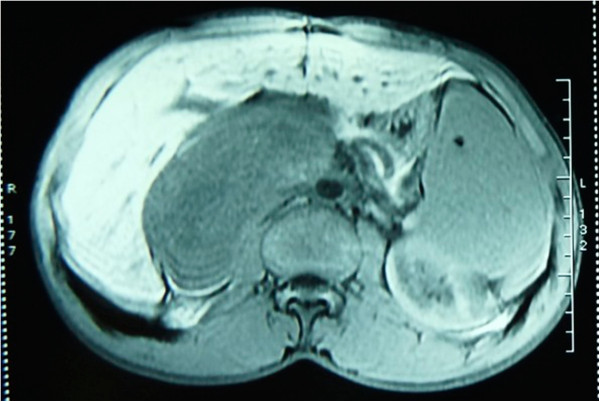
**T1-weighted enhanced MRI.**

**Figure 4:**
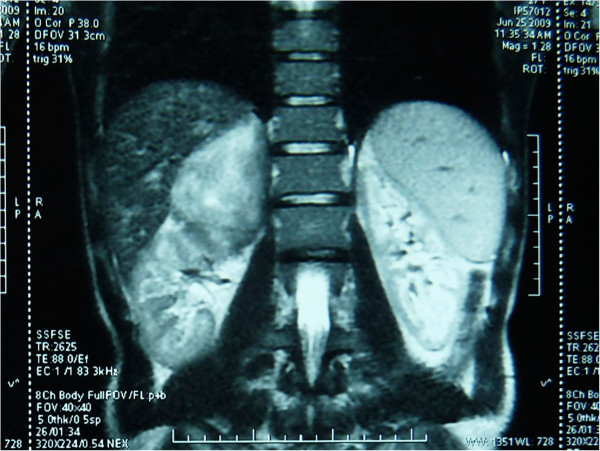
**T2-weighted MRI: coronal section.**

**Figure 5:**
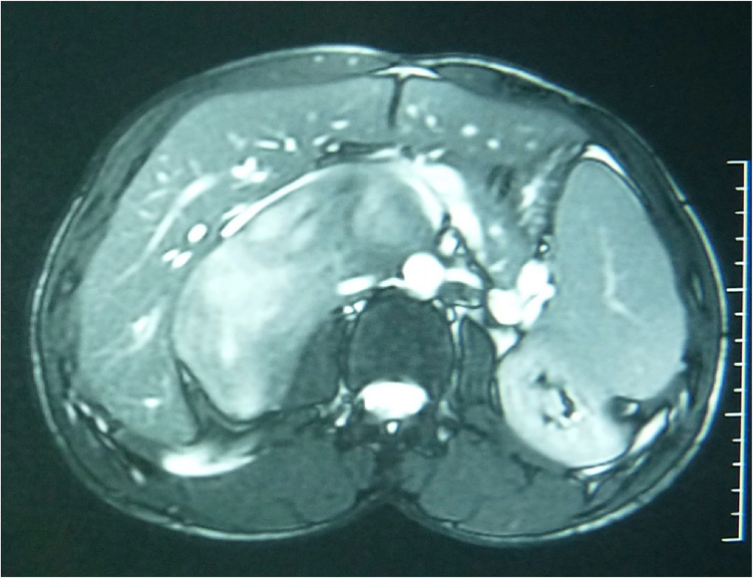
**T2-weighted MRI: axial section.**

**Figure 6:**
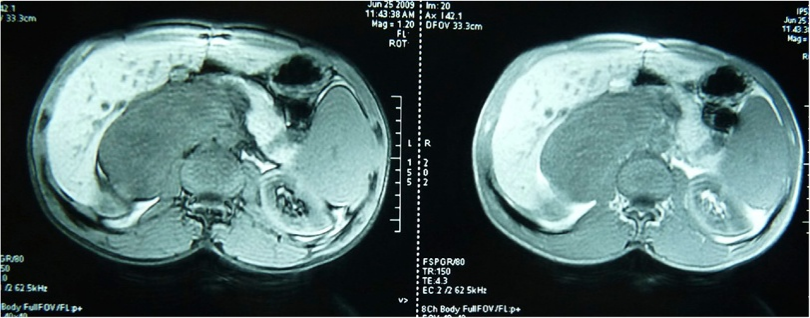
**MRI in–out phase.**

**Figure 7:**
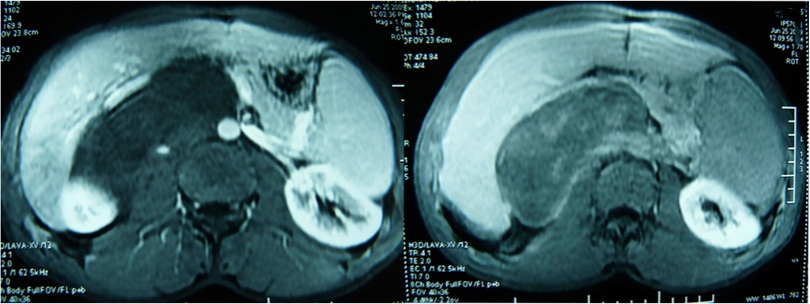
**MRI after intravenous administration of gadolinium.**

A 28-year-old Arabic man was referred to our unit after the discovery of a right adrenal incidentaloma during a workup for right subcostal pain. The patient had no other symptoms. Serum laboratory values included normal 24-hour urinary native catecholamine and metanephrine levels, normal adrenal corticotrophin and cortisol rhythms, normal 24-hour urinary free cortisol level, normal adrenal plasma aldosterone/plasma renin activity ratio, and normal androgen hormone levels. Computed tomography (CT) showed a well-circumscribed, homogeneous right adrenal tumor measuring 10 × 7 × 8 cm and exhibiting peripheral punctate calcifications. The density of this mass on unenhanced images was lower than that of muscle (Figure 1). Additionally, the mass was poorly enhanced with contrast medium (Figure 2). It was quite large, extending across the midline and behind the inferior vena cava. It surrounded the right renal artery and came into close contact with the left renal vein. On T1-weighted magnetic resonance imaging (MRI) (Figure 3), the tumor was visualized as a homogeneous mass with a signal intensity lower than that of the liver. On T2-weighted MRI (Figures 4 and 5), the lesion was heterogeneous with a signal intensity greater than that of the liver. No variations in the signal intensity were noted on chemical shift imaging (Figure 6). Poor and delayed tumor enhancement was observed after intravenous administration of gadolinium (Figure 7). Surgical resection was performed based on suspicion for a nonsecreting adrenal mass. Surgical exploration revealed a large right adrenal mass extending across the midline. The mass was completely resected. Pathological examination of the resected specimen revealed a mature GN.

## Conclusions

GN is an uncommon benign and mature neuroblastic tumor [1]. It is a nonmetabolic tumor that is usually located along the distribution of the sympathetic nervous system in the posterior mediastinum (41.5%), retroperitoneum (37.5%), or adrenal medulla (21.0%) [3, 4]. Adrenal GN is a rare tumor that occurs most frequently in the fourth and fifth decades of life; in contrast, GN of the retroperitoneum and posterior mediastinum often affect children. No sex predominance has been reported, but a familial disposition and an association with Turner syndrome and multiple endocrine neoplasia II have been identified [5]. Because adrenal GNs usually do not secrete catecholamines or other hormones, including steroid hormones [5, 6], they are often clinically silent and asymptomatic, even when large [6]. When symptomatic, the most frequent clinical features are abdominal pain or palpation of an abdominal mass.

Histopathologically, GN is entirely composed of ganglion cells and Schwannian stroma and does not contain neuroblasts, intermediate cells, or mitotic figures [4, 5]. The presence of neuroblasts indicates that the tumor is a ganglioneuroblastoma (GNB) or neuroblastoma (NB) and excludes GN as a differential diagnosis [4]. However, NB and GNB may mature to GN.

Most GNs are discovered incidentally on imaging studies [7, 8]. Ultrasonography shows a well-defined, homogeneous, hypoechogenic mass [4, 9]. CT usually reveals a homogeneous or slightly heterogeneous mass that tends to surround major blood vessels without compression or occlusion. The tumor is well-defined, hypodense, and poorly enhanced by contrast medium [4, 10]. Calcifications, typically fine and punctate, are seen in approximately 42–60% of GNs. Early enhancement of linear septae has been reported, and delayed heterogeneous contrast uptake has also been described in some cases. CT allows for an accurate description of the lesion and its relationship with vascular structures [11].

T1-weighted MRI shows a homogeneous mass with a signal intensity lower than that of the liver, and T2-weighted MRI shows a heterogeneous mass with a predominant signal intensity higher than that of the liver. T2-weighted MRI also shows no absolute change in signal intensity on chemical shift imaging. The high, heterogeneous signal intensity on T2-weighted images is presumed to be caused by a combination of the myxoid matrix and relatively low numbers of ganglion cells [4, 11]. Delayed and progressive enhancement of the mass is often observed after gadolinium administration. Neither early nor intense enhancement of GN is typically seen [4, 5, 9]. GN, like GNB and NB, may accumulate iodine-131-metaiodobenzylguanidine; thus, uptake of this agent does not allow for differentiation among these entities [4].

Preoperative diagnosis is difficult because of the lack of specific imaging findings [11, 12]. Percutaneous biopsy may help, but the presence of undifferentiated components limits its utility [10, 11]. The differential diagnosis of adrenal GN includes other adrenal solid masses such as pheochromocytoma, NB, GNB, adenoma, and carcinoma [2, 4, 11]. GNB and NB more frequently develop in children. They exhibit a higher degree of cellularity and a smaller extracellular space than does GN. This is the main cause of the higher unenhanced density on CT and a lower hypersignal on T2-weighted MRI. Calcifications are often punctuate in GN and amorphous in NB [11]. Pheochromocytoma secretes catecholamines, and this diagnosis is suspected in younger patients with hypertension. Cross-sectional imaging modalities reveal intense early enhancement in pheochromocytoma and progressive delayed enhancement in GN [2].

Certain biological and radiological features should lead the clinician to consider a diagnosis of GN: no elevated hormonal secretion; presence of punctuate calcifications, no vascular involvement, and nonenhanced attenuation of <40 Hounsfield units on CT; a homogeneous, hypointense mass on T1-weighted MRI; a heterogeneous, hyperintense adrenal mass on T2-weighted MRI; and poor, delayed enhancement on dynamic MRI.

In conclusion, adrenal GN is a diagnostic challenge on imaging studies [5]. This tumor is associated with various diagnostic problems because of its similarity to other solid adrenal tumors. However, some suggestive features on CT and MRI are helpful in achieving a correct diagnosis.

## Consent

Written informed consent was obtained from the patient for publication of this Case Report and any accompanying images. A copy of the written consent is available for review by the Editor-in-Chief of this journal.

## Abbreviations

CT: Computed tomography
MRI: Magnetic resonance imaging
GN: Ganglioneuroma
GNB: Ganglioneuroblastoma
NB: Neuroblastoma.

## References

[1] Kamoun M, Mnif MF, Rekik N, Belguith N, Charfi N, Mnif L, Elleuch M, Mnif F, Kamoun T, Mnif Z, Kamoun H, Sellami-Boudawara T, Hachicha M, Abid M (2010). Ganglioneuroma of adrenal gland in a patient with Turner syndrome. Annals of Diagnostic Pathology.

[2] Dunnick NR, Korobkin (2002). Imaging of adrenal incidentalomas: current status. Am J Roentgenology.

[3] Leavitt JR, Harold DL, Robinson RB (2000). Adrenal ganglioneuroma: a familial case. Urology.

[4] Lonergan GJ, Schwab CM, Suarez ES, Carlson CL (2002). Neuroblastoma, ganglioneuroblastoma, and ganglioneuroma: radiologic-pathologic correlation. Radiographics.

[5] Allende DS, Hansel DE, MacLennan GT (2009). Ganglioneuroma of the adrenal gland. J Urology.

[6] Qing Y, Bin X, Jian W, Li G, Linhui W, Bing L, Huiqing W, Yinghao S (2010). Adrenal ganglioneuromas: A 10-year experience in a Chinese population. Surgery.

[7] Cronin EM, Coffey JC, Herlihy D, Romics L, Aftab F, Keohane C, Redmond HP (2005). Massive retroperitoneal ganglioneuroma presenting with small bowel obstruction 18 years following initial diagnosis. Irish J Med Sci.

[8] Rha SE, Byun JY, Jung SE, Chun HJ, Lee HG, Lee JM (2003). Neurogenic tumors in the abdomen: tumor types and imaging characteristics. Radiographics.

[9] Yamaguchi K, Hara I, Takeda M, Tanaka K, Yamada Y, Fujisawa M, Kawabata G (2006). Two cases of ganglioneuroma. Urology.

[10] Zugor V, Schott GE, Kühn R, Labanaris AP (2009). Retroperitoneal ganglioneuroma in childhood–a presentation of two cases. Ped Neonatology.

[11] Otal P, Mezghani S, Hassissene S, Maleux G, Colombier D, Rousseau H, Joffre F (2001). Imaging of retroperitoneal ganglioneuroma. European Radiology.

[12] Maweja S, Materne R, Detrembleur N, de Leval L, Defechereux T, Meurisse M, Hamoir E (2007). Adrenal ganglioneuroma. Ame J Surgery.

